# Enhancing professional development in medical residency through a shadow curriculum: an evaluation based on Kirkpatrick model

**DOI:** 10.1186/s13104-025-07233-z

**Published:** 2025-04-20

**Authors:** Sahba Fekri, Amin Habibi, Hamed Khani, Samane Babaei, Masomeh Kalantarion

**Affiliations:** 1 Department of Ophthalmology, Labbafinejad Medical Center, Shahid Beheshti University of Medical Sciences, Tehran, Iran; 2 Department of Medical Education, School of Medical Education and Learning Technologies, Shahid Beheshti University of Medical Sciences, Tehran, Iran; 3 Department of Medical Education, Education Development Center (EDC), Smart University of Medical Sciences (SmUMS), Tehran, Iran

**Keywords:** Medical education, Program evaluation, Curriculum, Residency

## Abstract

**Introduction:**

Current medical residency programs often neglect critical areas of professional development, such as patient safety, stewardship, and effective clinical documentation. This study evaluates the effectiveness of a shadow curriculum designed to enhance these aspects for medical residents.

**Methods:**

The shadow curriculum was implemented for first-year residents in ophthalmology, internal medicine, and urology, consisting of an 8-hour workshop covering job encounters, stewardship, patient safety principles, medical documentation, and electronic prescribing. Conducted in 2023 with 22 residents, the evaluation utilized questionnaires, pre- and post-tests, and semi-structured interviews to assess satisfaction and learning outcomes.

**Results:**

54.55% of participants with a mean score of 23.66 (SD = 1.97) reported satisfaction with course content, and 63.63% with a mean score of 22.64 (SD = 2.63), were satisfied with the course organization. Pre/post-test results showed a significant knowledge increase (*p* < 0.001), particularly in antibiotic prescribing and patient safety. Qualitative interviews emphasized on three themes including consumer oriented learning, changing the perspective of teaching and learning, and promotion of self-directed learning.

**Conclusions:**

This study highlights the shadow curriculum’s effectiveness in improving residents’ professional satisfaction and knowledge. By prioritizing learner perspectives and extending opportunities beyond traditional settings, it fosters a personalized learning environment. These findings underscore the need to integrate shadow curricula into medical training to meet evolving educational needs and enhance professional development.

**Supplementary Information:**

The online version contains supplementary material available at 10.1186/s13104-025-07233-z.

## Introduction

Medical residency programs are vital for developing skills aligned with Accreditation Council for Graduate Medical Education (ACGME) competencies, preparing residents as advocates and caregivers. Updating educational methods is essential to meet evolving public healthcare needs [1, 2]. Surveys show that residents feel inadequately trained in professional development areas such as primary prevention, occupational exposures, stewardship, patient safety, and electronic documentation [3–6].

Given these gaps, the implementation of a shadow curriculum is crucial, where traditional curricula may not fully address the diverse learning needs of residents. Shadow education, also known as private supplementary tutoring [7–12], refers to various forms of supplemental learning experiences outside the formal curriculum, including classroom-based, small-group, and one-on-one tutoring [11–14]. This approach aims to enhance learners’ performance within formal educational systems and is characterized by its flexibility and alignment with individual abilities and interests. Unlike formal curricula, shadow education is supplementary and often provided by educational-commercial institutions to improve knowledge, behavior, and attitudes among learners [15, 16].

Research indicates that learners engaged in shadow education outperform their peers academically, enhancing performance across subjects. Its popularity arises from its alignment with individual abilities and interests, offering a flexible, tailored learning experience [10, 17]. The shadow education framework provides customized learning objectives independent of institutional curricula, fostering conscious learning opportunities that promote professional development [18]. This approach emphasizes personalized learning, allowing students to select subjects, mentors, and study pace, ultimately enhancing professional growth without overloading the formal curriculum [13, 19].

This evaluation study examines whether a shadow curriculum improves medical residents’ knowledge, self-directed learning, and engagement using the first and second levels of Kirkpatrick model.

## Method

### Setting and program description

Iran’s medical education system features a clinical residency of 3 to 6 years, training residents in disease prevention, diagnosis, and treatment. The shadow curriculum program seeks to update the residency curriculum, addressing societal needs and enhancing professional development while alleviating the formal curriculum’s burden.

In 2023, Labbafinejad Hospital implemented a shadow curriculum for residents, featuring an eight-hour workshop in job encounters, stewardship, patient safety, medical documentation, and electronic prescribing based on needs assessment of the vice chancellor, faculty members, and residents. An infectious disease specialist, an infection control specialist, a safety specialist, an occupational health specialist, and a health information technology specialist were empowered as course instructors.

### Participants and sampling

There were a total of 22 first-year residents of ophthalmology, urology, and internal medicine departments at Labbafinejad Hospital who were included through convenience sampling for quantitative evaluation of program. For the qualitative evaluation, a purposive sampling method was employed. Participants included residents who had experience in the training program, were willing to be interviewed, and possessed strong speaking skills. Interviewing 7 of them brought us to data saturation.

### Evaluation design and methods

An evaluation study design based on level 1 (reaction) and level 2 (learning) of Kirkpatrick model was employed for the quantitative and qualitative evaluation of this program. For this purpose, various methods and tools were utilized.

### Level 1: satisfaction (course content, course organization, and performance of instructors) and perspectives of residents

A standard satisfaction questionnaire approved by the Iranian Ministry of Health, which is currently used to evaluate continuing education programs, was used in this study. This questionnaire evaluates the training course in three dimensions: course content (5 questions), course organization (5 questions), and instructor performance (6 questions) (Supplementary file 1).

The content and course organization scores ranged from 5 to 25, with 5–15 deemed unacceptable and 16–25 acceptable. Content evaluation focused on practicality and job alignment, while course organization assessed communication methods and training equipment. Instructor performance was rated through six Likert scale questions, with 6–20 considered unacceptable and 21–30 acceptable.

The validity of the questionnaire was assessed using face and content validity. Face validity involved a qualitative review by a 4-member panel of experts from various fields (ophthalmology, internal medicine, and urology and medical education), leading to minor revisions for clarity and appropriateness. Content validity was quantitatively evaluated by calculating the CVR and CVI indices, based on feedback from 8 experts (medical education, instructional design, and clinical teachers). All 16 questions met the CVR threshold ≥ 0.75 (Lawshe’s table). The CVI results showed that all questions except questions 4 and 5 had scores higher than 0.79, and these two questions were slightly revised. Finally, to assess the reliability of the questionnaire, a pilot study was conducted with 10 residents, and the Cronbach’s alpha coefficient for the total instrument was calculated and confirmed to be 0.91.

Semi-structured interviews were conducted with residents at the program’s conclusion to gather perspectives. A qualified researcher led these interviews using a developed guide (Supplementary file 2), with informed consent. Each 20-minute telephone interview began with rapport-building questions, and qualitative data collection continued until theoretical saturation was achieved.

### Level 2: learning assessment

To evaluate learning, pre- and post-tests using MCQs measured residents’ knowledge. A committee of course instructors and specialists created 30 questions based on a test blueprint, ensuring face and content validity through a qualitative review session.

### Data analysis

To check the normality of the quantitative data, the Kolmogorov-Smirnov test was used, the results of which are presented in Table 1. A paired t-test was conducted, with quantitative data analyzed via SPSS version 21.

**Table 1:** Kolmogorov-Smirnov test to check the normality of the data

	*N*	Kolmogorov-Smirnov	*P*-value
Pre-test	22	0.682	> 0.05
Post-test	22	0.879	> 0.05

Qualitative data were analyzed through thematic content analysis per Graneheim and Lundman’s method [20]. Codes, subcategories, categories, and themes were extracted manually by the researchers from part to whole during an inductive process. To ensure rigor of the data, Guba and Lincoln’s trustworthiness criteria, credibility, dependability, confirmability and transferability, were followed through engagement with the data, peer check, member check and preparation of a comprehensive description of concepts, participants and methodology.

## Results

### Descriptive of participants

Table 2 shows demographic characteristics of the participants who completed the questionnaire and pre/post-test in quantitative evaluation and also demographics of interviewees in qualitative evaluation.

**Table 2:** Demographics of participants in quantitative and qualitative evaluation

Variables		Participants in quantitative evaluation *N* (%)	Participants in qualitative evaluation *N* (%)
**Gender**	Male Female	11 (50) 11 (50)	4 (57.15) 3 (42.85)
**Age**	26–36 37–47	15 (68.18) 7 (31.81)	5 (71.43) 2 (28.57)
**Residency field**	Ophthalmology Urology Internal medicine	8 (36.36) 6 (27.27) 8 (36.36)	3 (42.85) 2 (28.57) 2 (28.57)
Total		22 (100)	7 (100)

### Quantitative evaluation findings

Questionnaire scores on satisfaction with course content, course organization, and instructor performance indicated acceptable levels. Scores below average were deemed unacceptable.

In analysis of residents’ satisfaction, we found that 54.55% rated the course content as acceptable, with a mean score of 23.66 (SD = 1.97). Conversely, 45.45% deemed the course content unacceptable, resulting in a mean score of 14.1 (SD = 1.37). Regarding course organization, higher percentage (63.63%) of residents found it acceptable, with a mean score of 22.64 (SD = 2.63), while 36.37% rated it as unacceptable, yielding a mean score of 12.62 (SD = 1.99). These findings indicate a generally positive reception of both course content and course organization among residents.

Our findings indicate that Instructor 1, Instructor 2, and Instructor 3 each received an acceptable performance rating from 72.72% of residents, with 16 participants rating them as acceptable and 6 as unacceptable. Instructor 4 had an acceptable performance rating of 63.63%, with 14 residents finding it acceptable and 8 unacceptable. Finally, Instructor 5 received an acceptable performance rating of 68.18%, with 15 participants rating them as acceptable and 7 as unacceptable. These results reflect a generally positive perception of the instructors among residents.

Table 3 indicates a significant improvement in residents’ knowledge by subgroups (*p* < 0.001), particularly in antibiotic prescribing, stewardship, HBV/HIV/HCV prevention, patient safety, and medical record documentation after pre/post-tests.

**Table 3:** Average changes in pre/post-test scores by specialty

Specialty	Phase	*N*	Mean ± SD	*P*-value
Ophthalmology	Pre-test Post-test	8 8	17.50 ± 1.41 20.62 ± 2.50	< 0.001
Urology	Pre-test Post-test	6 6	11.83 ± 4.87 19.00 ± 2.09	< 0.01
Internal medicine	Pre-test Post-test	8 8	15.50 ± 1.77 18.87 ± 1.35	< 0.001

### Qualitative evaluation findings

Qualitative data from interviews were analyzed using content analysis. Participants’ responses were transcribed verbatim and reviewed multiple times. Relevant experiences were extracted and compiled, forming the unit of analysis. Meaning units were identified, coded, and organized into 14 sub-categories, 4 main categories, and 3 overarching themes.

**Table 4:** Themes, categories, and sub-categories emerging from interviews

Themes	Categories	Sub-categories
Consumer oriented learning	Hearing the voice of residents (learners) in education	Alignment of instructional materials and strategies with residents’ preferences and learning needs
		Pursuing experiences and learning opportunities to better address the desired learning needs and goals of residents
		Focusing on individual residents’ knowledge, attitudes, and skills needs
	Personalized learning and learning management	Creating an enriched and needs-based learning environment for residents
		Selection of instructors based on residents’ interests, needs, and accessibility
		Progressing in learning at a pace and rhythm suitable for individual learning
		Coverage of content and objectives beyond the prescribed or predicted curriculum in graduate and residency training
Changing the perspective of teaching and learning	Not attributing mere learning and academic progress to formal education	Providing content and guidelines to address cognitive, emotional, and psychological imbalances resulting from formal education among residents
		Remedial strategies to address residents’ knowledge, skill, and attitude gaps
		Preventing overreliance on formal education (formal curriculum and hidden) for the development of residents’ capabilities
		Coverage of content encompassing task-oriented objectives in assistantship training for job success
Promotion of self-directed learning	Creating internal motivation, commitment and responsibility in learning	Flexible instructional design and involvement of residents in determining learning objectives and content
		Quick progression from easy content and tasks to challenging content and tasks
		Promoting intrinsic value of learning over grade acquisition.

Table 4 outlines themes from interviews highlighting the program’s role in enhancing learning. It has been complemented by some resident statements which mention two subcategories of the qualitative analysis:

### Creating an enriched and needs-based learning environment for residents

Interviewee #1: “One day, I faced a sudden case that raised patient safety concerns. Previously, I hesitated to ask clinical professors questions, fearing judgment. However, the availability of instructors outside our clinical faculty encouraged me to seek clarification, ultimately helping prevent a major medical error and enhancing my understanding of patient safety.”

### Selection of instructors based on residents’ interests, needs, and accessibility

Interviewee #4: “I had direct access to course instructors, allowing me to seek help whenever needed. When I encountered a documentation issue, even my clinical professor was unsure. This highlighted the weaknesses in our regular training. Ultimately, the course benefited both residents and instructors.”

These statements highlight the positive impact and unique features of the program, emphasizing the shift in perspectives, improvement of learning outcomes, and enhancement of personalized learning in residency level.

## Discussion

Shadow education is a vital global phenomenon influencing learning today [8, 15, 21]. It is expanding globally, evolving into an alternative educational system that addresses diverse academic needs [7, 15, 22, 23]. It has evolved into an alternative educational system addressing academic challenges, serving as a vital supplement in various learning contexts [24]. It significantly impacts learning across countries, including South Korea, the United States, Japan, Bangladesh, Sri Lanka, India, and Canada [15, 25]. Although shadow education holds great importance [15, 26], it remains a 21st-century educational phenomenon with limited understanding [15]. In this study, a program was developed to address residents’ deficiencies in knowledge, skills, and attitudes, integrating shadow education into residency curriculum.

The study’s findings, based on the first level of the Kirkpatrick model, indicate a generally positive perception of both course content and course organization among residents, with higher satisfaction levels observed in the course organization aspect.

Additionally, evaluations of program instructors indicated satisfactory performance in delivering the educational program to a high standard. Overall, residents displayed a positive attitude towards the course. Since this program was developed based on a needs assessment conducted with the vice chancellor, faculty, and residents, it faced no resistance. Challenges regarding increased workloads were addressed by hiring non-clinical faculty as course instructors, consolidating workshops into one day, and adapting to residents’ schedules.

The evaluation revealed that the unique features of program enhanced residents’ learning and academic performance, categorized into three themes: consumer-oriented, changing educational perspectives, and promoting self-directed study habits. Shadow programs, involving informal learning through activities like physician shadowing and clinical experiences, significantly impact students’ professional development and shape their attitudes toward patients and the healthcare system [27].

The evaluation of the course’s effectiveness revealed significant knowledge improvement among residents, particularly in areas such as antibiotic prescribing, stewardship, prevention of HBV, HIV, and HCV post-needle stick, as well as patient safety principles and medical record documentation. This underscores the course’s positive impact on residents’ learning.

Developed shadow education in this study met the diverse educational needs of stakeholders by personalizing content based on residents’ specific knowledge, attitudes, and skills identified through needs assessments. Compared to traditional residency training, it enhanced self-directed learning through considering flexible instructional design and involvement of residents in determining learning objectives and content.

Shadow programs in medical education create personalized learning environments and tailored teaching strategies to enhance academic performance. These implicit concepts, not included in the formal curriculum, play a crucial role in shaping students’ professional development [28].

Generally, the implications of this evaluative research for medical education can be categorized and summarized as follows:

### Improving academic and occupational performance with an Understanding of the intrinsic value of learning

Learners engage in shadow education to improve academic and occupational performance [8, 12, 15]. Critics claim it prioritizes grades over intrinsic learning. However, the evaluated program emphasized professional development, indicating that a refined shadowing curriculum can enhance medical education.

### Consumer-based medical education

Shadow education meets the diverse educational needs of stakeholders by personalizing content based on residents’ specific knowledge, attitudes, and skills identified through needs assessments, thereby enhancing medical training effectiveness within standardized curricula and time constraints [7, 8, 15].

### Moving from monophonic discourse toward polyphonic discourse in medical education

A shadowing curriculum addresses learners’ needs through their feedback, enhancing program relevance and inclusivity by incorporating diverse stakeholder voices, applicable to both formal and hidden educational formats [29].

The shadow curriculum in medical education is poorly understood, often confused with hidden and informal curricula, complicating research and evaluation. Long course durations hinder data collection. Despite these challenges, this pioneering program introduces shadow education into medical training, necessitating further interventions and research to improve understanding and implementation.

This single-institution study limits the generalizability of its findings. The lack of a control group and short follow-up period raise concerns about attributing improvements solely to the shadow curriculum and the sustainability of gains. Additionally, potential confounding factors like prior resident experience and motivation should be acknowledged. Despite these limitations, this study is the first to explore the shadow curriculum concept in residency education, highlighting the need for further research.

## Conclusions

This study underscores the importance of consumer-based educational programs in medical education, particularly at the residency level, by highlighting learner perspectives. Findings reveal that shadow education enhances traditional curricula, increasing residents’ satisfaction and knowledge. To fully leverage its benefits, shadow education should be formally integrated into residency programs, ensuring structured implementation and alignment with educational goals. Future research should focus on developing frameworks for its incorporation into both formal and hidden curricula.

## Electronic supplementary material

Below is the link to the electronic supplementary material.

Supplementary Material 1

Supplementary Material 2

## Data Availability

No datasets were generated or analysed during the current study.
