# Supplementary material for: Enhancing professional development in medical residency through a shadow curriculum: an evaluation based on Kirkpatrick model
Source: BMC Res Notes. 2025 Apr 20;18:184. doi: 10.1186/s13104-025-07233-z (PMC12009517; doi:10.1186/s13104-025-07233-z)
Supplement: Supplementary file 1 — Supplementary Material 1 [file 13104_2025_7233_MOESM1_ESM.docx]

**Questionnaire**

**Course management:** Vice-Chancellor of Education and Research of Labbafinejad Hospital

**Venue:** Labbafinejad Hospital

Dear resident,

Improving the quality of training courses requires your cooperation. We would appreciate it if you could help us by providing your opinions in terms of course content and organization; and also to leave your feedback and suggestions in order to hold training courses as productively as possible.

Best regards

| **No** | **Item** | **Neutral** | **Poor** | **Moderate** | **Good** | **Very good** |
| --- | --- | --- | --- | --- | --- | --- |
| **A: Course content** | | | | | | |
| 1 | Applicability of material |  |  |  |  |  |
| 2 | New material |  |  |  |  |  |
| 3 | Quality and content of material |  |  |  |  |  |
| 4 | Full content coverage |  |  |  |  |  |
| 5 | Suitability of content with occupational tasks |  |  |  |  |  |
| **B: Course organization** | | | | | | |
| 6 | Course announcing and notification |  |  |  |  |  |
| 7 | Suitability of educational space |  |  |  |  |  |
| 8 | Suitability of educational equipment |  |  |  |  |  |
| 9 | Course schedule |  |  |  |  |  |
| 10 | Reception and service quality |  |  |  |  |  |
| **C: Instructor performance** | | | | | | |
| **Instructor 1** | | | | | | |
| Mastery of the material | |  |  |  |  |  |
| Ability to manage the classroom | |  |  |  |  |  |
| Engaging learners in discussion | |  |  |  |  |  |
| Answering questions | |  |  |  |  |  |
| Appropriate behavior | |  |  |  |  |  |
| Discipline | |  |  |  |  |  |
| **Instructor 2** | | | | | | |
| Mastery of the material | |  |  |  |  |  |
| Ability to manage the classroom | |  |  |  |  |  |
| Engaging learners in discussion | |  |  |  |  |  |
| Answering questions | |  |  |  |  |  |
| Appropriate behavior | |  |  |  |  |  |
| Discipline | |  |  |  |  |  |
| **Instructor 3** | | | | | | |
| Mastery of the material | |  |  |  |  |  |
| Ability to manage the classroom | |  |  |  |  |  |
| Engaging learners in discussion | |  |  |  |  |  |
| Answering questions | |  |  |  |  |  |
| Appropriate behavior | |  |  |  |  |  |
| Discipline | |  |  |  |  |  |
| **Instructor 4** | | | | | | |
| Mastery of the material | |  |  |  |  |  |
| Ability to manage the classroom | |  |  |  |  |  |
| Engaging learners in discussion | |  |  |  |  |  |
| Answering questions | |  |  |  |  |  |
| Appropriate behavior | |  |  |  |  |  |
| Discipline | |  |  |  |  |  |
| **Instructor 5** | | | | | | |
| Mastery of the material | |  |  |  |  |  |
| Ability to manage the classroom | |  |  |  |  |  |
| Engaging learners in discussion | |  |  |  |  |  |
| Answering questions | |  |  |  |  |  |
| Appropriate behavior | |  |  |  |  |  |
| Discipline | |  |  |  |  |  |
| **Positive points of the course:** | | | | | | |
| **Negative points of the course:** | | | | | | |
| **In your opinion, the content of this course will be effective in what part of your future occupation?** | | | | | | |
| **What other jobs do you think this course is suitable for?** | | | | | | |
