# Supplementary material for: Enhancing professional development in medical residency through a shadow curriculum: an evaluation based on Kirkpatrick model
Source: BMC Res Notes. 2025 Apr 20;18:184. doi: 10.1186/s13104-025-07233-z (PMC12009517; doi:10.1186/s13104-025-07233-z)
Supplement: Supplementary file 2 — Supplementary Material 2 [file 13104_2025_7233_MOESM2_ESM.docx]

**Semi-Structured Interview for the "Shadow Curriculum" Program**

**Introduction:**

Hello, thank you very much for taking the time to participate in this interview. The purpose of this interview is to explore your experiences with the "Shadow Curriculum" program and gather your feedback for its improvement.

Your responses will be confidential and used solely for research purposes.

- Can you describe your overall experience of participating in the "Shadow Curriculum" program?
- Do you feel that the educational materials and strategies of the program aligned with your needs?
- How did the program allow you to participate in determining the learning objectives and content of the program?
- How did the program address your learning needs and preferences?
- How did the program respond to your feedback, and did this feedback have any impact on improving the program?
- How did the program help you find appropriate learning experiences and opportunities that matched your learning needs and goals?
- How did the program help create a learning environment that was responsive to your needs?
- Did the program allow you to progress at a pace and rhythm that suited you?
- Were the selected mentors aligned with your needs and interests?
- Did the program help change your perspective on the concept of learning?
- Did the program help you enhance your self-directed learning?
- Did the program help you reduce your reliance on formal education and develop your capabilities?
- Did the program provide you with content that went beyond the prescribed curriculum?

Thank you for taking the time to answer these questions. Is there anything else you would like to add or any question you think should have been asked?

This interview will help us better understand and improve the program.
